# A database of freshwater fish species of the Amazon Basin

**DOI:** 10.1038/s41597-020-0436-4

**Published:** 2020-03-19

**Authors:** Céline Jézéquel, Pablo A. Tedesco, Rémy Bigorne, Javier A. Maldonado-Ocampo, Hernan Ortega, Max Hidalgo, Koen Martens, Gislene Torrente-Vilara, Jansen Zuanon, Astrid Acosta, Edwin Agudelo, Soraya Barrera Maure, Douglas A. Bastos, Juan Bogotá Gregory, Fernando G. Cabeceira, André L. C. Canto, Fernando M. Carvajal-Vallejos, Lucélia N. Carvalho, Ariana Cella-Ribeiro, Raphaël Covain, Carlos Donascimiento, Carolina R. C. Dória, Cleber Duarte, Efrem J. G. Ferreira, André V. Galuch, Tommaso Giarrizzo, Rafael P. Leitão, John G. Lundberg, Mabel Maldonado, José I. Mojica, Luciano F. A. Montag, Willian M. Ohara, Tiago H. S. Pires, Marc Pouilly, Saúl Prada-Pedreros, Luiz J. de Queiroz, Lucia Rapp Py-Daniel, Frank R. V. Ribeiro, Raúl Ríos Herrera, Jaime Sarmiento, Leandro M. Sousa, Lis F. Stegmann, Jonathan Valdiviezo-Rivera, Francisco Villa, Takayuki Yunoki, Thierry Oberdorff

**Affiliations:** 1 UMR EDB (Laboratoire Évolution et Diversité Biologique), CNRS 5174, IRD253, UPS, 118 route de Narbonne, F-31062 Toulouse, France; 2 Facultad de Ciencias, Pontificia Universidad Javeriana (PUJ), Unidad de Ecología y Sistemática (UNESIS), Laboratorio de Ictiología, Departamento de Biología, Carrera 7 No. 40–62, Bogotá, Colombia; 3 Museo de Historia Natural, Universidad Nacional Mayor San Marcos (MUSM), Departamento de Ictiología, Avenida Arenales 1256, Jesús María, 15072 Lima, Peru; 4 Royal Belgian Institute for Natural Sciences, OD Nature, Freshwater Biology, Vautierstraat 29, B-1000 Brussels, Belgium; 5 University of Ghent, Department Biology, K.L. Ledeganckstraat 35, B-9000 Gent, Belgium; 6 Universidade Federal de São Paulo, Departamento de Ciências do Mar, Campus Baixada Santista (UNIFESP). Rua Doutor Carvalho de Mendonça, 144, Encruzilhada, 11015-020 Santos, SP Brazil; 7 Instituto Nacional de Pesquisas da Amazônia (INPA), Coordenação de Biodiversidade, Avenida André Araújo, 2936, Petrópolis, 69067-375 Manaus, Amazonas Brazil; 8 Instituto Amazónico de Investigaciones Científicas Sinchi, Colección Ictiológica de la Amazonia Colombiana (CIACOL), Avenida Vasquez Cobo entre Calles 15 y 16, Leticia, Amazonas Colombia; 9 Museo Nacional de Historia Natural - MMAyA, Calle 26 de Cota Cota, La Paz, Bolivia; 10 Universidade Federal de Mato Grosso (UFMT), Campus Universitário de Cuiabá, Avenida Fernando Correa da Costa, 2367, 78060-900 Cuiabé, Mato Grosso Brazil; 11 Universidade Federal do Oeste do Pará (UFOPA), Instituto de Ciências e Tecnologia das Águas (ICTA), Rua Vera Paz, 68040-050 Santarém, Pará Brazil; 12 Universidad Mayor de San Simón, Unidad de Limnología y Recursos Acuáticos (UMSS-ULRA), Calle Sucre y parque la Torre, 2500 Cochabamba, Bolivia; 13 Universidade Federal de Mato Grosso (UFMT), Campus Universitário de Sinop, Avenida Alexandre Ferronato, 1200, 78550-728 Sinop, Mato Grosso Brazil; 14 Centro Universitário Aparício Carvalho, Departamento de Ciências Biológicas, Rua das Ararás, 241, 76811-678 Porto Velho, Rondônia Brazil; 15 Muséum d’histoire naturelle (MHNG), Département d’herpétologie et d’ichtyologie, route de Malagnou 1, case postale 6434, CH-1211 Genève, Switzerland; 16 Instituto Alexander von Humboldt (IAvHP), Calle 28A#15-09, Bogota, Colombia; 17 Universidade Federal de Rondônia, Departamento de Biologia (UNIR), Campus José Ribeiro Filho, Rodovia BR-364, s/n km 9,5, 76801-059 Porto Velho, Rondônia Brazil; 18 Universidade Federal do Pará (UFPA), Núcleo de Ecologia Aquática e Pesca da Amazônia (NEAP), Avenida Perimetral, 2651, 66077-830 Belém, Pará Brazil; 19 Universidade Federal de Minas Gerais (UFMG), Departamento de Genética, Ecologia e Evolução, Instituto de Ciências Biológicas, Avenida Antonio Carlos, 6627, 31270-901 Belo Horizonte, Minas Gerais Brazil; 20 Academy of Natural Sciences of Philadelphia and Drexel University (ANSP), Ichthyology Department, 1900 Benjamin Franklin Parkway, Philadelphia, PA 19103 USA; 21 Instituto de Ciencias Naturales, Universidad Nacional de Colombia (UN ICN-MHN), Ak 30#45-03, Bogota, Colombia; 22 Universidade Federal do Pará (UFPA), Ecology and Conservation Lab, Rua Augusto Correa, 01, 66075-110 Belém, Pará Brazil; 23 Universidade Federal de Rondônia (UNIR), Laboratório de Ciências Ambientais, Campus Presidente Médici, Rua da Paz, 4376, 76916-000 Presidente Médici, Rondônia Brazil; 24 Laboratoire de Biologie des Organismes et Ecosystèmes Aquatiques, Muséum National d’Histoire Naturelle, CNRS, IRD, SU, UCN, UA, 43 Rue Cuvier, F-75005 Paris, France; 25 University of Geneva, Department of Genetics and Evolution (UNIGE GenEv), Boulevard D’Yvoy 4, 1205 Genève, Switzerland; 26 Instituto para la Investigación y la Preservación del Patrimonio Cultural y Natural (INCIVA), Calle 6#24-80, Avenida Roosevelt, Cali, Colombia; 27 Universidade Federal do Pará (UFPA), Laboratório de Ictiologia de Altamira, Rua Coronel José Porfírio, 2515, 68372-040 Altamira, Pará Brazil; 28 Instituto Nacional De Biodiversidad (INABIO), Pje Rumipamba 341 y Avenida de los Shyris (Parque La Carolina), 170150 Quito, Ecuador; 29 Universidad del Tolima (UT CZUT-IC), Facultad de Ciencias, Grupo de Investigación en Zoología, Barrio Santa Helena Parte Alta, Ibagué, Tolima Colombia; 30 Universidad Autónoma del Beni, Centro de Investigación de Recursos Acuáticos (CIRA), Avenida 6 De Agosto No. 61, Trinidad, Bolivia

**Keywords:** Freshwater ecology, Tropical ecology, Biodiversity

## Abstract

The Amazon Basin is an unquestionable biodiversity hotspot, containing the highest freshwater biodiversity on earth and facing off a recent increase in anthropogenic threats. The current knowledge on the spatial distribution of the freshwater fish species is greatly deficient in this basin, preventing a comprehensive understanding of this hyper-diverse ecosystem as a whole. Filling this gap was the priority of a transnational collaborative project, *i.e*. the AmazonFish project - https://www.amazon-fish.com/. Relying on the outputs of this project, we provide the most complete fish species distribution records covering the whole Amazon drainage. The database, including 2,406 validated freshwater native fish species, 232,936 georeferenced records, results from an extensive survey of species distribution including 590 different sources (*e.g*. published articles, grey literature, online biodiversity databases and scientific collections from museums and universities worldwide) and field expeditions conducted during the project. This database, delivered at both georeferenced localities (21,500 localities) and sub-drainages grains (144 units), represents a highly valuable source of information for further studies on freshwater fish biodiversity, biogeography and conservation.

## Abstract (summary)

**Table:** 

Measurement(s)	Diversity • Fish • spatial pattern
Technology Type(s)	digital curation
Factor Type(s)	geographic location
Sample Characteristic - Organism	fish
Sample Characteristic - Environment	drainage basin
Sample Characteristic - Location	Amazon Basin

Machine-accessible metadata file describing the reported data: 10.6084/m9.figshare.11920800

## Background & Summary

The Amazon Basin covers more than 6,000,000 km^2^, produces about 20% of the world’s freshwater discharge^1–3^ and contains the highest freshwater richness on Earth^4^. This is especially true for Amazonian fishes that represent ~15% of all freshwater fish species described worldwide^5,6^. The processes having generated this highly diverse fish fauna are incompletely understood. However, low rates of species extinction over several millions of years due to the diversity in aquatic habitats and the stability in favourable climatic conditions are most probably involved^7,8^. Compared to other large riverine ecosystems on Earth, the Amazon Basin and its fish fauna are still in a relatively good state of conservation^9,10^. Nevertheless, recent expansion of infrastructures and economic activities are likely to endanger this fish fauna in the near future due to the substantial increase in threats such as habitat fragmentation and river flow modification by dams, deforestation, roads, mining, urban and/or agricultural pollutions, species introduction and overfishing^11^. Climate change will probably exacerbate these threats further amplifying changes in the structure and function of fish communities^11,12^.

Our knowledge on fish species occurrence and spatial distribution within the Amazon Basin is far from complete. Numerous new species are described each year^13,14^ and some large areas are still unknown in several portions of the basin^15,16^. This was among the key motivations of the transnational collaborative project AmazonFish (https://www.amazon-fish.com/) that aimed to compile the most complete and up-to-date information currently available on freshwater fish species distribution for the entire Amazon drainage basin and to initiate scientific collecting expeditions in under-sampled areas to fill the gaps. This database is thus the result of mobilizing information available from various sources (published articles, grey literature, field expedition reports, online biodiversity databases and scientific collections from museums and universities worldwide) and field expeditions organized during the project. This compilation, covering a time span of almost two hundred years (1834–2019), currently comprises 2,406 valid native freshwater fish species recorded from 590 different sources representing more than 235,064 occurrence records (232,936 georeferenced and 2,128 non-georeferenced) and 21,500 sampled localities (hereafter called sampling sites). Two parallel compilation efforts on the distribution of freshwater fish species in the Amazon Basin have been recently released^17,18^. The field guide book from van der Sleen and Albert^17^ delivers a general view of the current knowledge of fish ecology and distribution maps at the genus level only. The compilation from Dagosta & De Pinna^18^ provides species lists for 30 Amazonian sub-drainages but suffers from a lack of information^19^. Here, we complement and refine these previous initiatives by providing species-level distributions on a database format combining available information at both sampling site and sub-drainage grains (144 units).

By compiling the knowledge on the spatial distribution of freshwater fishes and addressing the taxonomic and sampling gaps, the Amazon Fish database should become a valuable and long-lasting source of information for ecological and conservation studies. The database is currently being used to analyse fish diversity patterns at the Amazon Basin scale^19^, to evaluate the potential effect of climate change^20^ and fragmentation^21^ on this biodiversity and to define diversity hotspots for the whole basin conservation priorities^22^. Besides improving our fundamental knowledge of the patterns and processes involved in the generation of Neotropical freshwater fish diversity, the information provided can also help developing regional conservation programs and contributing to largescale transnational ecosystem management initiatives.

Species occurrences are delivered here at two spatial grains, sampling sites (with precise geographic coordinates) and sub-drainage (144 units) grains. The database is organised in two sub-datasets and one shapefile. The first dataset contains the species list by sub-drainage with the taxonomic FishBase reference name (Family, Genus, Referent species valid name and Author), the species status (‘native’ or ‘exotic’) and the occurrence species status (‘valid’, ‘to be verified’, ‘marine’; see Technical validation for more details). The second dataset contains the geographic coordinates for the georeferenced records, the information source of each record, and the original name of the species cited in the source (‘synonym’, ‘typing error’). Finally, the shapefile delineates all the sub-drainages, along with the corresponding geographic information (*e.g*. main river name, main country, geographic coordinates and surface area of the sub-drainage). The database is obviously not complete, regular updates are planned in the future to include new occurrence records from literature, collections and new field expeditions planned to cover sampling gaps, together with the distribution of newly described species and nomenclatural changes.

## Methods

### Information sources

The database results from the transnational collaborative project AmazonFish (ERANetLAC/DCC-0210) whose purpose was to identify and compile all known information sources available on freshwater fish species occurrences for the entire Amazon drainage basin. The original project included researchers from (1) the French Institute for Development (IRD) in France, (2) the Pontificia Universidad Javeriana (PUJ-UNESIS) in Colombia, (3) the Museo de Historia Natural de la Universidad Nacional Mayor de San Marcos (MUSM) in Peru and (4) the Royal Belgian Institute of Natural Sciences in Belgium. The project also benefited from official collaborations with researchers from Brazil (Instituto Nacional de Pesquisas da Amazônia INPA; Universidade Federal de São Paulo UNIFESP; Universidade Federal de Rondônia UNIR; Universidade Federal do Pará UFPA; Universidade Federal do Oeste do Pará UFOPA; Universidade Federal de Mato Grosso UFMT), Colombia (Instituto Alexander von Humboldt IAvH, Universidad Nacional de Colombia UN ICN-MHN, Universidad del Tolima UT-CZUT, Instituto Amazónico de Investigaciones Científicas SINCHI-CIACOL, Instituto para la Investigación y la Preservación del Patrimonio Cultural y Natural del Valle del Cauca INCIVA, Universidad Católica de Oriente UCO), Ecuador (Museo Ecuatoriano de Ciencias Naturales MECN-DP, Instituto Nacional De Biodiversidad INABIO), Bolivia (Universidad Mayor de San Simon UMSS-ULRA, Colección Boliviana de Fauna MNHN–IE UMSA, Universidad Autónoma del Beni CIRA) and Switzerland (Museum d’Histoire Naturelle de Genève, MHNG). All these partners brought into the project, besides their Neotropical fish taxonomic expertise needed to produce a high-quality database, existing fish databases from their own collections and expeditions, and a large networking capacity that was essential for identifying and involving other data providers.

In order to build the AmazonFish database, an inventory of the possible data sources was conducted at the beginning of the project in early 2016 and data from a wide range of sources were compiled and standardized in a single dataset.

The information used includes five source types:

A. Information extracted from the literature (published articles, books, grey literature)
B. Data from online biodiversity databases (*i.e*. GBIF and others)
C. Data from museums and universities collections
D. Data held or compiled by the project partners (*e.g*. country level)
E. New data from sampling campaigns organized within the framework of the project

An inventory of all the literature sources (published articles, books, technical reports) existent for the Amazon Basin led to more than 800 different documents that were subsequently analysed, from which 459 provided valuable data on fish species distribution, not redundant with any official collection. An important amount of data was extracted from the most used and frequently updated online biodiversity databases (see details in Table 1). These repositories release biological data under a Creative Commons licence in which the user agrees to acknowledge the data sources. Data from museums and universities collections not available through these online facilities were obtained by contacting the curators or researchers in charge and integrating them as official project collaborators (curators and researchers mainly from Brazil, Ecuador and Bolivia). The project partners (Colombia and Peru) compiled data at the country level. For Colombia^23,24^, the data were previously published through the GBIF network. For Peru, the AmazonFish project has supported the numeric digitalization of the national freshwater fish collections^25,26^, which is still an ongoing work (51% of the records have been digitalized so far). Finally, supplementary occurrence data were obtained during five sampling campaigns in Brazil, Colombia and Peru and targeting under-sampled areas identified during the project.

**Table 1:** Online Biodiversity Repository Sources with the complete name, the number of occurrences and institutions, the last consulted date and the internet link.

Biodiversity Repository	Online Biodiversity Repository complete name	Number of occurrences	Number of institutions	Last consulted date	Internet link
GBIF	Global Biodiversity Information Facility	84,507	60	1/5/2020	https://www.gbif.org/
SpeciesLink	SpeciesLink Portal, Brazil	38,656	21	1/5/2020	http://splink.cria.org.br
Fishnet2	Fishnet2 Portal, USA	32,133	26	9/27/2016	http://fishnet2.net/
iDigBio	Integrated Digitized Biocollections	21,021	24	4/10/2019	https://www.idigbio.org/
ICMBio	Instituto Chico Mendes de Conservaçao da Biodiversidade, Brazil	9,054	1	10/31/2019	https://portaldabiodiversidade.icmbio.gov.br/portal/
AMNH	American Museum of Natural History, New York, USA	223	1	9/27/2016	https://www.amnh.org/research/vertebrate-zoology/ichthyology
SiBBr	Sistema de Informaçao sobre a Biodiversidade Brasileira, Brazil	130	6	9/27/2016	https://www.sibbr.gov.br/
IABIN	Inter-American Biodiversity Information Network	39	1	9/27/2016	https://www.oas.org/en/sedi/dsd/iabin/default.asp

### Species, taxonomy and status

All occurrences not identified to species level were discarded (*i.e*. occurrences giving only genus names commonly abbreviated to sp., species affinis commonly abbreviated to: sp. aff., aff., or affin. or species confer abbreviated to cf.). All species scientific names are reported in the database as appearing in each information source and were carefully checked for typing errors and misspellings. Because taxonomy is a ‘moving target’, species names were standardized and linked to an internationally accepted standardized name and associated taxonomic information in order to find synonymies and provide accepted names. All species names were first searched in FishBase through the ‘rfishbase’ package^27^ from the R environment^28^ allowing to easily obtain the valid species names. For species names absent from FishBase, a manual search was applied in the Eschmeyer’s Catalog of Fishes (http://researcharchive.calacademy.org/research/ichthyology/catalog/fishcatmain.asp). This last step allowed finding valid names and recently described species not yet included in FishBase. The final standardized species list contains 3,366 valid species names avoiding biases due to synonyms and uncertain identifications (see ‘Technical Validation’). We also integrated all remaining species names, *i.e*. not listed in any of the two scientific catalogues, as ‘unknown name at present’ (294 species names).

A species status (‘native’ or ‘exotic’) and an occurrence species status (‘valid’, ‘to be verified’ or ‘marine’) were assigned to each species. The species status distinguishes ‘native’ from ‘exotic’ species (*i.e*. non-native species introduced in the Amazon Basin)^5^ and the occurrence species status is divided in three criteria: (1) ‘valid’ (species known to belong to the Amazon Basin); (2) ‘to be verified’ (species whose presence in the Amazon Basin is not certain because of possible mis-identification or localisation errors); and (3) ‘marine’ (species whose primary habitat is not freshwater, based on information available in FishBase or Eschmeyer’s Catalog of Fishes).

At this time, the database contains 2,406 ‘native’ and ‘valid’ freshwater fish species, 837 ‘to be verified’ species, 105 ‘marine’, 18 ‘exotic’ and 294 ‘unknown’ species. The species considered as ‘native’ and ‘valid’, *i.e*. freshwater species belonging to the Amazon Basin, were the only species considered in all species numbers reported below.

### Sub-drainages delineation

**Fig. 1:**
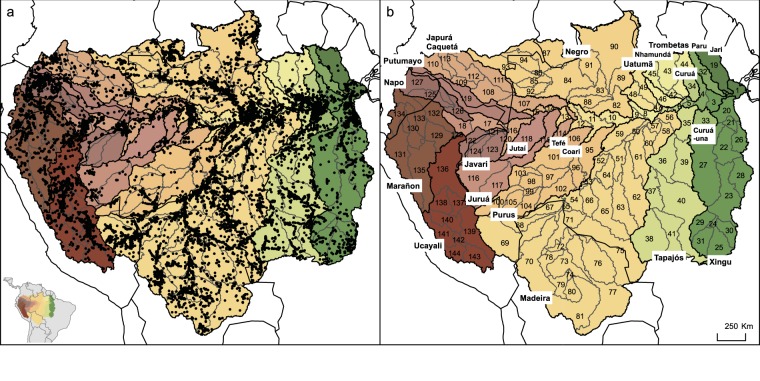
(**a**) Distribution of sampling sites recorded in the AmazonFish database and (**b**) delimitation and codes of the 144 sub-drainages units (see corresponding names in Online-only Table 1), based on a modified version of HydroBASINS (see methods). The major tributaries of the Amazon Basin are represented in different colours and their names are added in bold.

The Amazon Basin was defined here as the area of land where precipitation collects and drains off into a common outlet. This excludes de facto the Tocantins basin and Guiana coastal streams (see Fig. 1), but constitutes for freshwater fishes an ideal grain for conducting biogeographical and/or macroecological studies^29^.

The hydrological sub-drainage units within the Amazon Basin were delineated using the HydroBASINS framework, a subset of the HydroSHEDS database^30^. The levels 5 and 6 were combined with a constraint area of >20,000 km^2^, at the exception of sub-drainages located in the river mainstem where delineation was based on the distance between two main tributaries entering the mainstem. This led to obtain a total of 144 sub-drainages covering the entire Amazon system (Fig. 1).

## Data Records

The database^31^ provides a comprehensive overview of the current knowledge of the fish species diversity and distribution in the Amazon Basin, with 21,500 sites (Fig. 1), 232,936 georeferenced occurrence records and 2,128 non-georeferenced records from 590 different sources combining literature, scientific collections, sampling campaigns and partner’s datasets. Some of the online biodiversity repositories (Table 1) showed some redundancies because often referring to the same collections. In this specific case, only one occurrence record was retained.

The main sources of the database are online biodiversity databases (56% of the occurrences), followed by locally hosted data from the scientific partners (Peru and Colombia), museums and universities from Brazil, Bolivia and Ecuador (38%), literature data (5% of the records) and data obtained during sampling campaigns by partners from Colombia, Peru and Brazil (1%). This represents 93 different collections from Scientific Institutions, 31 Partners references, 459 literature references and five AmazonFish expeditions.

The database includes information for 56 families, 514 genera and 2,406 native valid freshwater species, virtually half of the *circa* 4,760 total number of species known for the whole Neotropical biogeographic region^5,6^. Among these 2,406 species, 1,402 are found exclusively in the Amazon Basin (*i.e*. species appearing nowhere else on Earth; Amazonian endemic species) based on the global species distribution provided by Tedesco *et al*.^5^.

**Fig. 2:**
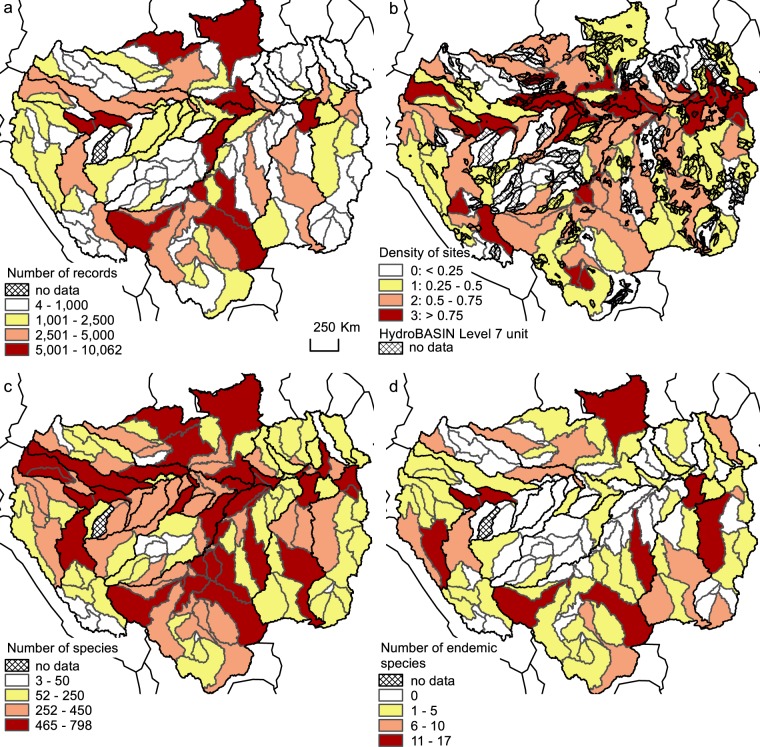
(**a**) Number of records, (**b**) density of sites (number of sites divided by the sub-drainage area and areas without information using the HydroBASINS Level7 spatial grain unit^30^), (**c**) total number of species and (**d**) number of endemic species (species present only in the Amazon Basin and only in the sub-drainage) for the 144 sub-drainage units.

The lowland Amazon and its two main tributaries, the Negro and Madeira Rivers regroup the highest number of sites, occurrences and the highest diversity (Table 2), whereas less information is available for some small tributaries. At the sub-drainage grain, the density of sites presents an important spatial variability (Online-only Table 1 and Fig. 2). For instance, the Curuçá sub-drainage belonging to the Javari River, currently lacks information about its ichthyofauna. The ‘updates and limitations’ section below presents a more detailed overview of the spatial data gaps.

**Table 2:** Summary table with the total number of sites, occurrences and total number of families, genera, species and endemic species (i.e. Amazon endemic species present only in the sub-drainage) for each Amazon major tributary (with their surface area in km^2^).

Major Tributary	Area	Number of sites	Number of occurrences	Number of Families	Number of Genera	Number of species	Number of endemic species
Amazonas	100,687	1,463	16,365	51	342	971	11
Jari	58,207	80	471	41	160	227	9
Xingu	511,169	1,701	13,215	50	314	821	73
Paru	39,289	10	28	13	20	22	1
Curuá-una	31,116	123	1,025	38	119	195	2
Curuá	25,291	42	189	25	58	80	—
Tapajós	492,570	1,804	17,765	52	334	982	66
Trombetas	126,619	243	2,883	47	237	494	5
Nhamundá	28,609	108	630	36	129	235	1
Uatumã	67,920	174	1,692	47	191	416	—
Madeira	1,496,852	5,514	73,048	52	404	1,406	135
Negro	711,562	2,566	27,983	53	393	1,233	83
Solimões	186,654	1,856	23,732	51	362	1,113	35
Purus	377,158	631	9,310	48	319	836	5
Coari	35,588	59	795	46	189	323	—
Japurá	268,954	793	6,399	51	314	838	12
Tefé	24,269	230	2,226	48	245	545	2
Juruá	188,895	293	3,337	47	256	557	1
Jutaí	78,109	96	1,354	43	175	322	—
Putumayo	120,579	282	3,282	48	287	705	2
Javari	108,966	150	3,140	48	254	552	1
Napo	102,190	639	6,832	48	298	744	9
Marañon	363,296	1,088	8,356	49	286	747	39
Ucayali	352,304	1,555	11,007	49	289	727	28
Amazon Basin	5,896,853	21,500	235,064	56	514	2,406	1,402

The whole dataset is organised in three sub-sets^31^: a table of the species list by sub-drainage (‘GeneralDistribution’), a table of occurrence records with sources (‘CompleteDatabase’), and a shapefile of the 144 sub-drainages (‘SubDrainageShapefile’).

The first sub-set (‘GeneralDistribution’) contains the species list by sub-drainage with the taxonomic reference name (Family, Genus, Referent species valid scientific name and Author), the species status (‘native’ or ‘exotic’) and the occurrence species status (‘valid’, ‘to be verified’, ‘marine’). The corresponding table has nine columns (see Table 3).

**Table 3:** Detailed legend of the information given in each column of the 2 datasets (9 columns for the GeneralDistribution file and 22 columns for the CompleteDatabase file).

		Column Name	Description
General Distribution	Complete Database	Family.Referent.Species	Family name of the referent species
		Genus.Referent.Species	Genus name of the referent species
		Referent.Species.Name	Referent species: valid scientific species name (FishBase or Eschmeyer’s Catalog of Fishes) at the time of releasing the database
		Author.Referent.Species	Author’s name of the referent species
		Species.Status	Species status: ‘native’, ‘exotic’ or ‘unknown name at present’
		Occurrence.Status	Occurrence species status for native species: ‘valid’, ‘to be verified’ or ‘marine’
		Major.Tributary.Name	Major Amazon Tributary name
		SubDrainage.Name	Unique name of the sub-drainage
		SubDrainage.Code	Unique code of the sub-drainage
		Original.Species.Name.Source	Original species name in the source
		Species.State	State of the original species name: ‘synonym’ or ‘typing error’
		Longitude.X	Longitude coordinates (3 decimal rounded)
		Latitude.Y	Latitude coordinates (3 decimal rounded)
		Data.Source	Data source: ‘AmazonFish Expedition’, ‘Literature’, ‘Online Biodiversity Database’ or ‘Partners Datasets’
		Biodiversity.Repository	Biodiversity Repository source for the ‘Online Biodiversity Database’ source. The combination of two or more repositories mean that the museum or university collection is available from different sources: GBIF, SpeciesLink, FishNet2, iDigBio, AMNH, SiBBr, IABIN, see Table 1
		Institution.Code	Scientific Institution Code
		Institution.Name	Scientific Institution complete name
		GBIF.Citation	GBIF Citation of the Scientific Institution
		GBIF.DOI	GBIF DOI of the Scientific Institution
		Literature.Reference	Complete literature reference (Author, Date, Title and Scientific Journal)
		Partners.Citation	Citation reference of the Partner dataset
		Non.Georeferenced	Occurrences non-georeferenced: Sub-drainage Information (species occurrence information at the sub-drainage grain), Approximated Coordinates (species occurrence information at river or reach scales), Geographic Error (given coordinates of the site do not correspond to the sub-drainage information of the source)

The second sub-set (‘CompleteDatabase’) provides the geographic coordinates for the georeferenced sampling sites and the information source of each record. It is complemented with the original name of the species cited in the source (‘synonym’ or ‘typing error’) and those species with status ‘unknown name at present’. The detailed sources contain the source type of the data (‘Literature’, ‘Online Biodiversity Database’, ‘Partners Datasets’ and ‘AmazonFish Expedition’), the Biodiversity Repository source for the Online Biodiversity Database, the Scientific Institution Code and complete name, the GBIF Citation and DOI, the complete literature reference and the citation reference of the Partner dataset. Finally, the non-georeferenced occurrences are separated in three categories, ‘sub-drainage information’ (species occurrence information at the sub-drainage grain), ‘approximated coordinates’ (species occurrence information at river or reach scales) and ‘geographic error’ (the geographical coordinates of a site do not correspond to the geographical location given in the source). The corresponding table has 22 columns (see Table 3).

The third sub-set (‘SubDrainageShapefile’), corresponds to shapefile delineating all the sub-drainages and their corresponding geographic information and is organized in eight columns: (1) the major Amazon tributary name (MTRIB_NM), (2) the unique major Amazon tributary code (MTRIB_CD), (3) the unique sub-drainage name (SBD_NM), (4) the unique sub-drainage code (SBD_CD), (5) the surface area of the sub-drainage (AREA, in km^2^), (6) the main country where it belongs (COUNTRY), (7,8) the centroid longitude and latitude coordinates of the sub-drainage (CENT_X and CENT_Y).

The two table sub-sets (‘GeneralDistribution’ and ‘CompleteDatabase’) are in CSV format (columns separated by commas) and the shapefile sub-set (‘SubDrainageShapefile’) in ArcGis SHP format^31^. Both formats can be linked to the species occurrence table using the unique sub-drainage code or name to visualize and analyse species distribution using any adapted software (*e.g*. R or QGIS, http://qgis.osgeo.org). The sampling coordinates and shapefile are in the World Geodetic System 1984 (WGS84) datum and geographic coordinate system. The files of the database are in ‘CSV’ format (UTF-8 encoding, comma separator) and can be uploaded by most statistical software, spreadsheets or any other database management systems. The current version of the database can be retrieved from Figshare^31^, the AmazonFish website (https://www.amazon-fish.com/) and the Freshwater Biodiversity data portal (https://data.freshwaterbiodiversity.eu/).

## Technical Validation

### Taxonomic and status validation

Each species name found in a given information source was confronted to the valid and synonym species names lists from FishBase and Eschmeyer’s Catalog of Fishes to ensure the identifications validity provided by the information source. This taxonomic validation identified 1,332 synonyms, 781 typing errors and 294 unknown species names (names not listed in any of the two scientific catalogues). The original scientific names of the species are reported in the expanded table of the database (‘CompleteDatabase’), where users can extract sub-species, synonyms or unknown species names.

After having validated the taxonomic names, we further verified the presence certainty in the Amazon Basin of all the taxonomically valid species recorded in our database. This careful review was an essential step in the elaboration of the database and resulted in assigning a status to each species. The species status is based on the information provided by the data source, expert opinion from the AmazonFish partners and information about the species general distribution available in FishBase or Eschmeyer’s Catalog of Fishes catalogues. When the presence of a taxon was inconsistent with its actual known distribution, the species was classified as ‘to be verified’. A recently published database on the global distribution of freshwater fish species^5^ was also consulted to verify the overall distribution of each species, their exotic status and to identify species endemic to the Amazon Basin.

As a result, the database provides not only information on the validity of each species, but also on species occurrences and names that need further attention (‘to be verified’ and ‘unknown name at present’). This gives the opportunity for database users to refer to their own expertise and knowledge to validate or not the accuracy of the original source, species name and distribution (ideally, giving feedback to the AmazonFish project, https://www.amazon-fish.com/).

### Species distribution validation

The geographic coordinates of the sites were compared to the location name of the sub-basin given in the source. In case of mismatch, the coordinates were removed from the database and the information was kept only at the sub-basin grain and referenced as ‘geographic error’.

The geographic accuracy of the species distribution (for ‘native’ and ‘valid’ species) inside the Amazon Basin was checked using a basic geographic analysis. A convex hull envelop was delineated for each species based on its occurrence points, resulting in a list of sub-drainages potentially occupied by a given species. This list was then compared to the list of sub-drainages where the species had at least one record. From this comparison, *circa* 200 species showed some inconsistent distributions (outlying occurrences). All these occurrences were consequently carefully checked and further validated or excluded (see ‘ExcludedOccurrences’ file^31^).

### Updates and limitations

The database is obviously not complete and definitive, and we aim to keep the high-quality level of the database with regular updates, ideally with bi-annual steps, depending on human and financial resources. More than 100 new fish species were described between 2017 and 2019, which makes this update effort crucial in order to improve our knowledge about the distribution of freshwater fish within the Amazon Basin. The technical and taxonomic validation procedures described above will be applied to any new information included in the database. Three main factors will be considered in future updates: (1) new or previously non-available data sources with species lists or records; (2) occurrences of newly described species; and (3) nomenclature changes in the taxonomic classification.

If the main rivers of the Amazon Basin appear well surveyed, some gaps do exist, however in various parts of the basin (Fig. 2). These gaps are mainly located in zones either difficult to access due to the topography and/or located in protected areas (indigenous lands or protected areas). Identifying never-sampled (to our knowledge) or under-sampled sub-drainages is a first step to guide increasing sampling efforts in these areas. The AmazonFish project has already initiated this process, by supporting the numeric digitalization of the national freshwater fish collections from Peru^25,26^ and by initiating sampling campaigns in detected gaps in Colombia, Peru and Brazil. All these spatial gaps in the database will also be prioritized in future updates through literature and web-based sources checking. Researchers holding fish distribution data from any of the current gaps or under-sampled areas (Fig. 2) and that wish to share these data are welcome to join the project. This information will be included with the complete source, after validation, in the next update of the database.

## Online-only Table

**Online-only Table 1:** List of the 144 sub-drainage units with total number of sites, occurrences, families, genera, species and endemics (i.e. Amazon endemics present only in the sub-drainage) for each sub-drainage.

Major Tributary	Sub-drainage code	Sub-drainage name	Area	Number of sites	Number of occurrences	Number of Families	Number of Genera	Number of species	Number of endemic species
Amazonas	1	Amazon1	15,948	96	849	37	110	176	2
Amazonas	2	Amazon2	2,655	64	410	17	48	65	—
Amazonas	3	Amazon3	21,135	130	1,051	44	185	312	—
Amazonas	4	Amazon4	21,969	246	3,401	49	245	540	3
Amazonas	5	Amazon5	5,915	129	1,409	47	200	381	—
Amazonas	6	Amazon6	5,729	163	1,967	48	259	533	2
Amazonas	7	Amazon7	3,992	19	450	40	128	209	—
Amazonas	8	Amazon8	17,154	260	3,344	48	263	597	2
Amazonas	9	Amazon9	6,190	356	3,484	47	248	555	—
Solimões	10	Amazon10	22,036	420	6,428	48	272	616	—
Solimões	11	Amazon11	32,871	120	1,339	43	179	309	1
Solimões	12	Amazon12	17,459	152	1,154	47	196	353	—
Solimões	13	Amazon13	11,906	160	1,028	41	170	278	—
Solimões	14	Amazon14	4,105	70	650	37	150	224	—
Solimões	15	Amazon15	8,626	143	1,020	39	154	227	—
Solimões	16	Amazon16	29,147	98	876	41	193	332	—
Solimões	17	Amazon17	32,795	350	6,002	49	283	723	11
Solimões	18	Amazon18	27,709	343	5,235	47	287	682	17
Jari	19	Jari	58,207	80	471	41	160	227	9
Xingu	20	Xingu1	31,315	558	3,861	48	263	550	1
Xingu	21	Xingu2	10,125	323	3,772	44	224	491	8
Xingu	22	Xingu3	48,113	182	1,050	39	142	237	—
Xingu	23	Xingu4	78,902	101	633	34	103	160	2
Xingu	24	Xingu5	4,770	3	4	3	3	3	—
Xingu	25	Xingu6	44,731	89	460	39	109	182	6
Xingu	26	Bacajá	25,642	43	573	38	119	189	1
Xingu	27	Iriri	141,746	206	1,703	42	184	365	15
Xingu	28	Fresco	43,785	68	409	28	64	103	1
Xingu	29	Manissauá - Miçu	28,015	53	385	22	45	83	—
Xingu	30	Suiá-Missu	23,705	39	152	20	43	67	—
Xingu	31	Ronuro	30,320	36	213	29	63	106	—
Paru	32	Paru	39,289	10	28	13	20	22	1
Curuá-una	33	Curuá-una	31,116	123	1,025	38	119	195	2
Curuá	34	Curuá	25,291	42	189	25	58	80	—
Tapajós	35	Tapajós1	42,528	585	6,380	51	291	703	11
Tapajós	36	Tapajós2	59,069	192	2,549	45	192	373	—
Tapajós	37	Juruena1	38,454	71	713	29	78	134	1
Tapajós	38	Juruena2	93,687	238	1,791	35	94	198	8
Tapajós	39	Jamanxim	58,520	89	841	42	163	320	2
Tapajós	40	Teles Pires	141,420	486	4,652	45	197	470	10
Tapajós	41	Arinos	58,892	143	839	32	75	157	1
Trombetas	42	Trombetas1	7,054	186	2,305	46	212	426	3
Trombetas	43	Trombetas2	51,860	33	359	32	116	172	1
Trombetas	44	Paru do Oeste	41,917	18	157	30	84	119	—
Trombetas	45	Mapuera	25,788	7	62	22	47	57	—
Nhamundá	46	Nhamundá	28,609	108	630	36	129	235	1
Uatumã	47	Uatumã1	3,480	16	305	43	130	215	—
Uatumã	48	Uatumã2	29,947	114	984	40	143	291	—
Uatumã	49	Jatapu	34,493	44	403	39	94	169	—
Madeira	50	Madeira1	9,293	278	3,128	47	246	479	—
Madeira	51	Madeira2	31,133	78	907	45	206	358	—
Madeira	52	Madeira3	19,105	103	1,501	45	241	465	—
Madeira	53	Madeira4	3,644	49	1,404	42	205	370	—
Madeira	54	Madeira5	24,509	505	6,087	48	295	640	—
Madeira	55	Madeira6	5,262	83	943	42	190	353	—
Madeira	56	Andira1	21,770	35	442	42	122	210	—
Madeira	57	Andira2	10,725	53	593	41	120	186	—
Madeira	58	Maués	26,329	75	665	42	116	192	1
Madeira	59	Luna	45,792	143	2,111	47	240	519	—
Madeira	60	Abacaxis	22,114	21	347	31	74	115	—
Madeira	61	Canuma	52,208	51	514	38	133	222	—
Madeira	62	Aripuanã	87,229	311	3,696	50	271	587	12
Madeira	63	Roosevelt	59,330	50	362	35	98	154	1
Madeira	64	Marmelos	27,456	57	590	40	128	212	—
Madeira	65	Jiparaná	74,738	421	10,062	48	251	596	4
Madeira	66	Candeias	29,265	89	1,411	45	243	485	1
Madeira	67	Abunã	31,970	58	527	38	147	255	—
Madeira	68	Orthon	33,576	198	4,425	42	193	385	1
Madeira	69	Madre de Dios	128,843	735	6,666	46	256	610	17
Madeira	70	Beni	118,948	312	2,759	46	215	443	4
Madeira	71	Mamoré1	45,675	226	3,207	46	243	523	4
Madeira	72	Mamoré2	19,246	46	878	38	154	258	—
Madeira	73	Mamoré3	43,774	170	3,009	40	184	342	2
Madeira	74	Mamoré4	3,260	11	38	11	26	29	—
Madeira	75	Guaporé - Iténez	151,316	520	7,649	45	269	688	13
Madeira	76	Blanco Baures	76,639	138	3,158	40	182	374	1
Madeira	77	Itonamas	125,073	158	2,127	42	182	364	7
Madeira	78	Yacuma	21,386	37	806	36	142	232	—
Madeira	79	Isiboro	21,046	169	1,318	38	144	209	1
Madeira	80	Chapare	20,139	135	1,084	38	143	243	1
Madeira	81	Grande	106,059	199	634	32	90	147	2
Negro	82	Negro1	52,502	676	8,431	49	304	798	2
Negro	83	Negro2	36,700	294	2,078	45	210	420	2
Negro	84	Negro3	91,495	305	3,027	45	241	591	10
Negro	85	Negro4	18,934	134	1,162	44	168	328	2
Negro	86	Negro5	3,061	12	75	26	51	64	—
Negro	87	Negro6	88,274	362	4,440	44	222	487	5
Negro	88	Unini	27,310	67	802	45	145	268	—
Negro	89	Jauaperí	38,882	26	163	29	68	105	3
Negro	90	Branco	189,945	401	5,645	50	309	754	14
Negro	91	Demini	39,592	64	479	41	130	230	1
Negro	92	Marié	25,266	6	15	5	7	11	—
Negro	93	Vaupés	64,085	214	1,613	38	153	326	8
Negro	94	Içana	35,516	5	53	18	37	52	—
Purus	95	Purus1	61,569	300	5,614	48	283	632	1
Purus	96	Purus2	26,028	67	854	42	161	261	—
Purus	97	Purus3	29,525	6	45	18	33	39	—
Purus	98	Purus4	22,944	5	24	15	20	23	—
Purus	99	Purus5	5,691	10	72	10	23	34	—
Purus	100	Purus6	36,639	50	494	32	108	156	—
Purus	101	Tapaua	62,893	25	187	29	77	108	—
Purus	102	Ituxi	43,748	18	161	27	50	73	—
Purus	103	Pauini	25,128	9	49	17	29	35	—
Purus	104	Acre	36,526	126	1,423	43	171	322	3
Purus	105	Iaco	26,467	15	387	34	105	149	—
Coari	106	Coari	35,588	59	795	46	189	323	—
Japurá	107	Japurá	62,545	423	3,499	48	273	579	6
Japurá	108	Caquetá1	44,315	57	683	41	141	271	—
Japurá	109	Caquetá2	14,182	34	331	37	113	183	—
Japurá	110	Caquetá3	33,591	197	1,229	40	147	297	6
Japurá	111	Apaporis	56,490	44	430	38	108	189	—
Japurá	112	Yarí	36,650	23	133	20	51	70	—
Japurá	113	Caguán	21,181	15	94	18	31	35	—
Tefé	114	Tefé	24,269	230	2,226	48	245	545	2
Juruá	115	Juruá1	53,073	156	1,768	44	211	377	—
Juruá	116	Juruá2	81,109	124	1,412	41	188	327	1
Juruá	117	Envira	54,713	13	157	29	71	86	—
Jutaí	118	Jutaí	78,109	96	1,354	43	175	322	—
Putumayo	119	Putumayo	120,579	282	3,282	48	287	705	2
Javari	120	Javari1	1,391	39	590	43	157	260	1
Javari	121	Javari2	6,315	53	1,186	46	206	358	—
Javari	122	Javari3	32,845	27	399	34	119	191	—
Javari	123	Ituí	43,215	31	965	45	186	315	—
Javari	124	Curuçá	25,200	0	0	—	—	—	—
Napo	125	Curaray	26,586	66	1,327	48	284	599	3
Napo	126	Napo1	22,578	48	649	41	153	267	—
Napo	127	Napo2	53,026	525	4,856	46	243	509	4
Marañon	128	Marañon1	2,213	20	166	29	79	108	—
Marañon	129	Marañon2	40,458	50	677	39	151	232	2
Marañon	130	Marañon3	31,657	80	811	41	166	293	1
Marañon	131	Marañon4	81,150	180	1,191	34	115	236	7
Marañon	132	Tigre	42,905	219	1,752	39	150	280	2
Marañon	133	Pastaza	42,271	207	1,633	42	182	339	2
Marañon	134	Santiago	32,951	139	1,116	35	121	210	3
Marañon	135	Huallaga	89,691	193	1,010	39	141	252	12
Ucayali	136	Ucayali1	110,606	552	4,949	47	269	607	10
Ucayali	137	Ucayali2	21,956	19	91	17	50	66	—
Ucayali	138	Pachitea	28,908	235	1,530	35	131	229	1
Ucayali	139	Urubamba	59,359	536	3,764	34	125	248	2
Ucayali	140	Tambo	32,277	126	529	27	79	124	4
Ucayali	141	Mantaro	34,535	51	75	6	6	11	1
Ucayali	142	Apurimac1	6,875	4	11	2	6	6	—
Ucayali	143	Apurimac2	34,568	18	35	5	12	14	—
Ucayali	144	Pampas	23,220	14	23	4	10	12	—
